# Association analysis in a Latin American population revealed ethnic differences in rheumatoid arthritis-associated SNPs in Caucasian and Asian populations

**DOI:** 10.1038/s41598-020-64659-0

**Published:** 2020-05-12

**Authors:** P. Castro-Santos, R. A. Verdugo, R. Alonso-Arias, M. A. Gutiérrez, J. Suazo, J. C. Aguillón, J. Olloquequi, C. Pinochet, A. Lucia, L. A. Quiñones, R. Díaz-Peña

**Affiliations:** 1 Facultad de Ciencias de la Salud, Universidad Autónoma de Chile, Talca, Chile; 2 Inmunología, Centro de Investigaciones Biomédicas (CINBIO), Universidad de Vigo, Vigo, Spain; 3 Programa de Genética Humana, ICBM, Facultad de Medicina, Universidad de Chile, Santiago, Chile; 4 Departamento de Oncología Básico Clínica, Facultad de Medicina, Universidad de Chile, Santiago, Chile; 5 Immunology Department, Hospital Universitario Central de Asturias, Oviedo, Spain; 6 Rheumatology, Almirante Nef Naval Hospital, Viña del Mar, Valparaíso, Chile; 7 Valparaíso University, Viña del Mar, Valparaíso, Chile; 8 Instituto de Investigación en Ciencias Odontológicas, Facultad de Odontología, Universidad de Chile, Santiago, Chile; 9 Immune Regulation and Tolerance Research Group, Programa de Inmunología, ICBM, Facultad de Medicina, Universidad de Chile, Santiago, Chile; 10 Hospital Regional de Talca, Talca, Chile; 11 Universidad Europea de Madrid (Faculty of Sports Sciences) and Research Institute Hospital 12 de Octubre (‘i + 12’), Madrid, Spain; 12 Laboratory of Chemical Carcinogenesis and Pharmacogenetics, Department de Basic-Clinical Oncology, Faculty of Medicine, University of Chile, Santiago, Chile; 13 Latin American Network for Implementation and Validation of Clinical Pharmacogenomics Guidelines (RELIVAF-CYTED), Madrid, Spain

**Keywords:** Genetic markers, Rheumatoid arthritis, Risk factors

## Abstract

Large genome-wide association studies (GWAS) have increased our knowledge of the genetic risk factors of rheumatoid arthritis (RA). However, little is known about genetic susceptibility in populations with a large admixture of Amerindian ancestry. The aim of the present study was to test the generalizability of previously reported RA *loci* in a Latin American (LA) population with admixed ancestry. We selected 128 single nucleotide polymorphisms (SNPs) in linkage equilibrium, with high association to RA in multiple populations of non-Amerindian origin. Genotyping of 118 SNPs was performed in 313 RA patients/487 healthy control subjects by mid-density arrays of polymerase chain reaction (PCR). Some of the identified associations were validated in an additional cohort (250 cases/290 controls). One marker, the SNP rs2451258, located upstream of T Cell Activation RhoGTPase Activating Protein (*TAGAP*) gene, showed significant association with RA (p = 5 × 10^−3^), whereas 18 markers exhibited suggestive associations (p < 0.05). Haplotype testing showed association of some groups of adjacent SNPs around the signal transducer and activator of transcription 4 (*STAT4*) gene (p = 9.82 × 10^−3^ to 2.04 × 10^−3^) with RA. Our major finding was little replication of previously reported genetic associations with RA. These results suggest that performing GWAS and admixture mapping in LA populations has the potential to reveal novel loci associated with RA. This in turn might help to gain insight into the ‘pathogenomics’ of this disease and to explore trans-population differences for RA in general.

## Introduction

Rheumatoid arthritis (RA) is an autoimmune inflammatory rheumatic disease that affects mainly synovial joints among many tissues and organs. It affects approximately 1% of the population worldwide^1^ and, although this condition can develop at any age, RA affects women more frequently than men and is mainly diagnosed between the ages of 40–60 years. In Latin America (LA), differences towards women seem to be higher, whereas prevalence has been estimated between 0.2–0.5%^2,3^. In Chile, there are data showing that the overall prevalence of RA based on clinical examination is 0.46%^4^.

The etiology of RA is multifactorial and partially unknown because of the complex interactions between genetic and environmental factors. Approximately 50% of RA risk is thought to be genetic, and one-third of this risk is associated with the human leukocyte antigen (HLA) locus^5^, specifically *HLA-DRB1* shared alleles (SE), which encode a common amino acid sequence^6^. Since 2007 about 101 RA risk *loci* have emerged from genome-wide association studies (GWAS) and subsequent GWAS meta-analyses^7,8^, mostly in individuals from European and/or Asian populations (Supplementary Table 1). In fact, none of the GWAS pertaining to RA has been performed in LA populations (Supplementary Table 1).

It is generally accepted that many common risk variants are shared between multiethnic populations, but allele frequencies of disease-associated single nucleotide polymorphisms (SNPs) vary significantly among ethnic groups due to genetic drift or selection^9^. Linkage between causal variants and tag SNPs included in genotyping microarrays might vary depending on population-specific pattern of recombination which in turn, is largely affected by population size, founder effects and admixture processes. In addition, populations with different histories may carry distinct causal mutations even in similar loci. All of these factors can preclude generalization of genetic associations from one population to another, and suggest testing for locus- or haplotype-wise rather than SNP-wise generalization^10^.

López Herráez *et al*.^11^ examined susceptibility loci for RA in LA populations. In this study, a strong association with HLA region was observed, with three independent effects, probably due to the diverse origin of the patients (Argentina, Mexico, Chile, and Peru). Some of the RA associations previously reported in GWAS were also replicated in the study by López Herráez and coworkers, but with moderate significant values (including protein tyrosine phosphatase, non-receptor type 22 (lymphoid) [*PTPN22*] and signal transducer and activator of transcription 4 [*STAT4*] genes). However, in general, genetic association studies on RA have not been robustly replicated in LA populations. Therefore, the aim of the present study was to carry out a high-density SNP genotyping in candidate genes to test their association with susceptibility to RA in the Chilean population, in order to provide insight on the cross-ethnic generalizability of known European and Asian RA risk *loci* to LA populations.

## Results

In the present study, five hundred and sixty-three (42.0%) of the included individuals suffered RA. Supplementary Table 2 shows the characteristics of the RA patients that were used for the analysis. The mean age was 48 and 58 years for cohort 1 and 2, respectively, and 84.7% and 81.0% of the patients were women. The mean duration of the disease was 8 years. Anti-cyclic citrullinated peptide (CCP) antibodies were determined in a total of 218 patients being positive in 164 of them (75.23%), whereas rheumatoid factor (RF) was determined in 300 patients being positive in 264 (88.0%). The RA group did not differ from the control group with regard to any of the clinical parameters included in the study (data not shown).

The present findings do not show replicable association of individual SNPs with RA. Among 128 SNPs genotyped, 118 passed all the quality filters, after excluding SNPs with a minor allele frequency <0.01 or missingness > 0.1 and those that were not in Hardy-Weinberg equilibrium (HWE) (p < 0.001) (Supplementary Table 3). Only two markers (2%) showed significant associations (p ≤ 0.01): rs1635567 and rs2469434 (Table 1), of which none was confirmed in Cohort 2. When data from both cohorts were combined, rs2469434 was still significant whereas rs1635567 could not be tested because the assay failed in Cohort 2. However, the combined analysis revealed a new significant association for rs2451258 (combined p = 5 × 10^−3^; p = 0.09 after Bonferroni correction for multiple testing) (Table 1). Eighteen markers exhibited suggestive associations (p < 0.05), whereas the associations of the remainder of SNPs included in the study were not significant. The significantly-associated SNPs in peptidyl arginine deiminase, type IV (*PADI4*), Protein tyrosine phosphatase, non-receptor type 22 (*PTPN22*), signal transducer and activator of transcription 4 (*STAT4*), cytotoxic T-lymphocyte-associated protein 4 (*CTLA4*), tumor necrosis factor, alpha-induced protein 3 (*TNFAIP3*), and chemokine receptor 6 (*CCR6* genes), identified in Caucasian and Asian populations, were not replicated in the Chilean population (Supplementary Fig. 1).

**Table 1:** Association analysis of the replicated SNPs as single markers in cohort 1 and cohort 2 and the joint analysis (only SNPs with P ≤ 0.1 are shown).

CHR	SNP	POSITION	Gene	A1	Cohort 1			Cohort 2			Joint analysis		
					MAF		P	MAF		P	MAF		Combined P
					RA Patients	Controls		RA Patients	Controls		RA Patients	Controls	
1	rs1635567^a^	17683041	*PADI4*	C	0.33	0.41	0.005						
18	rs2469434	67544046	*CD226*	C	0.32	0.26	0.009	0.31	0.29	0.52	0.32	0.27	0.01
1	rs2477134	17633572	*PADI4*	G	0.37	0.31	0.016	0.38	0.35	0.34	0.37	0.32	0.03
7	rs3778753 ^a^	128580042	*IRF5*	C	0.34	0.28	0.018						
21	rs9979383	36715761	*RUNX1*	C	0.31	0.25	0.018	0.29	0.28	0.82	0.31	0.27	0.04
11	rs4409785	95311422	*CEP57*	C	0.16	0.12	0.029	0.14	0.13	0.63	0.15	0.12	0.04
2	rs3024903	191895607	*STAT4*	T	0.15	0.11	0.031	0.13	0.11	0.30	0.14	0.11	0.05
22	rs3218251	37545505	*IL2RB*	A	0.18	0.14	0.037	0.18	0.13	0.03	0.18	0.14	0.02
6	rs2451258	159506600	*TAGAP*	C	0.15	0.19	0.038	0.14	0.22	0.003	0.15	0.20	0.005
20	rs4239702 ^a^	44749251	*CD40*	T	0.22	0.18	0.044						
8	rs998731	81095395	*TDP52*	C	0.36	0.31	0.051	0.33	0.34	0.88	0.36	0.32	0.04
10	rs2275806	8095340	*GATA3*	A	0.38	0.43	0.052	0.38	0.44	0.08	0.38	0.43	0.02
1	rs2843401	2528133	*MMEL1*	T	0.41	0.46	0.058	0.40	0.43	0.28	0.40	0.45	0.02
4	rs2664035	48220839	*TEC*	A	0.39	0.44	0.064	0.39	0.38	0.70	0.39	0.42	0.16
2	rs10175798	30449594	*LBH*	G	0.33	0.37	0.068	0.34	0.35	0.88	0.33	0.37	0.11
11	rs73013527	128496952	*ETS1*	T	0.36	0.41	0.073	0.36	0.39	0.43	0.36	0.41	0.04
2	rs11889341	191943742	*STAT4*	T	0.42	0.38	0.074	0.43	0.38	0.09	0.43	0.38	0.02
14	rs1950897	68760141	*RAD51B*	G	0.21	0.25	0.078	0.20	0.23	0.29	0.20	0.24	0.05
16	rs4780401	11839326	*TNXNDC11*	T	0.40	0.35	0.079	0.39	0.34	0.07	0.39	0.35	0.05
2	rs11571302	204742934	*CTLA4*	G	0.52	0.47	0.088	0.52	0.46	0.07	0.47	0.52	0.02
20	rs6032662	44734310	*CD40*	C	0.21	0.18	0.091	0.20	0.20	0.92	0.20	0.18	0.14
1	rs2228145	154426970	*IL6R*	A	0.42	0.38	0.093	0.43	0.40	0.32	0.44	0.39	0.02
17	rs12936409	38043649	*IKZF3*	A	0.46	0.42	0.1	0.45	0.41	0.27	0.46	0.42	0.07

CHR = chromosome; SNP = single-nucleotide polymorphism; A1 = minor allele nucleotide; RA = rheumatoid arthritis; and MAF = minor allele frequency.

^a^Failed in the replication phase (genotyping of the cohort 2).

**Figure 1:**
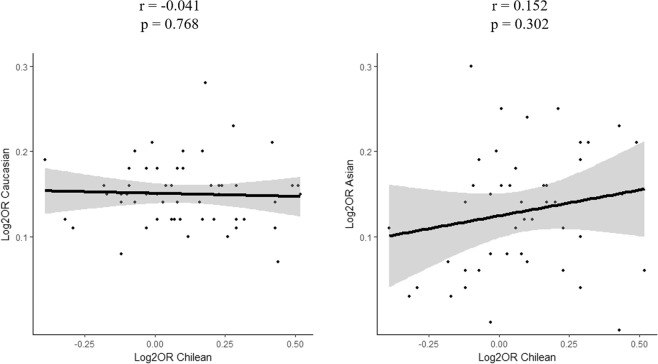
Correlation between log(odds ratio) from data published in GWA studies carried out in Caucasian an Asian population versus log(odds ratio) reported in this study (7). OR = odds ratio; GWA = genome association analysis. The respective regression lines with the Pearson correlation’s r-values are indicated.

**Figure 2:**
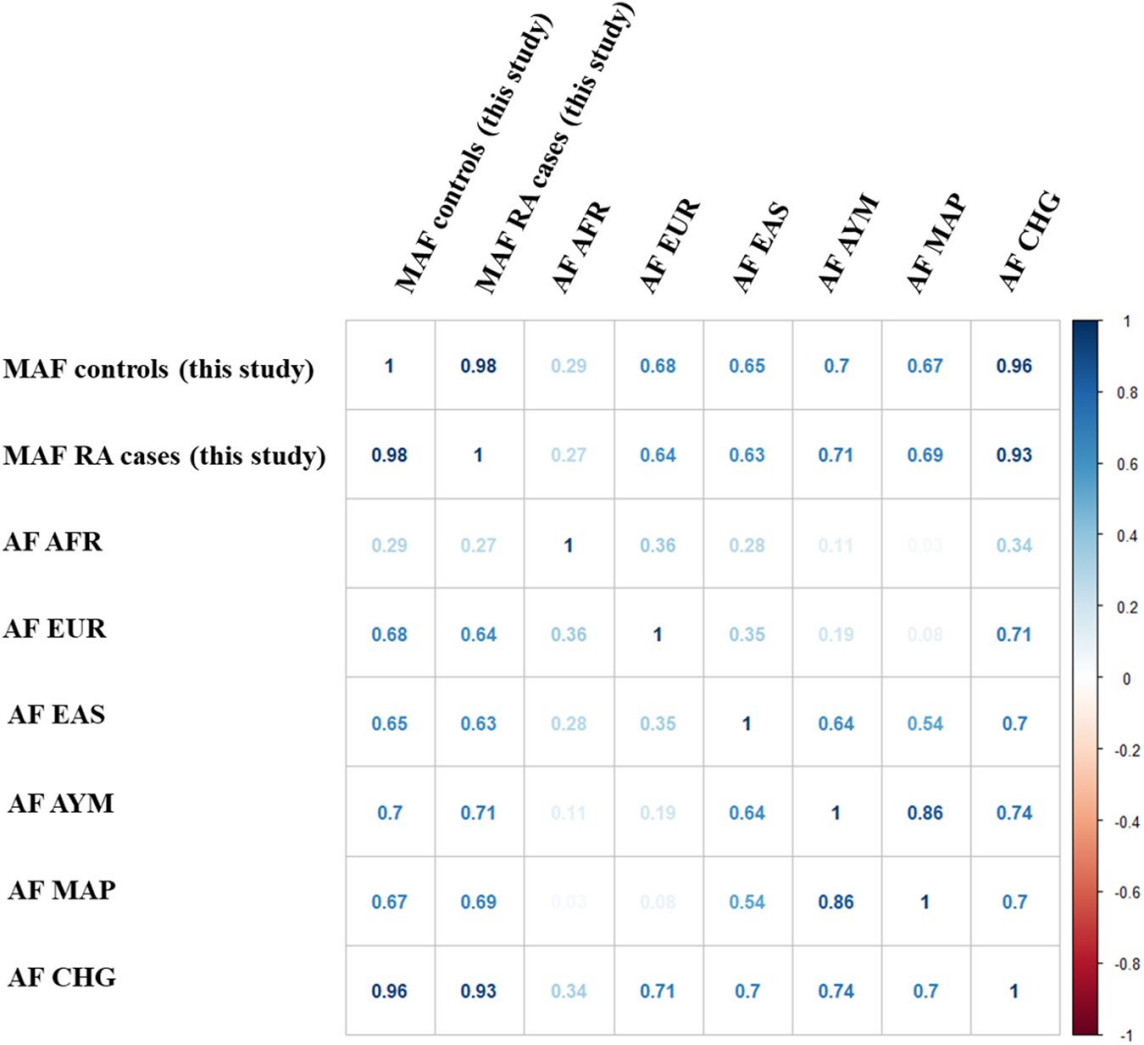
Correlation matrix between allele frequencies of the SNPs analyzed in different populations. MAF = minor allele frequency; AF = allele frequency; AFR = African; EUR = European; EAS = East Asian; AYM = Aymara; MAP = Mapuche; CHG = ChileGenomico.

We next determined the correlation between odds ratio (OR) derived from our study and OR previously reported in GWAS from Caucasian and Asian population^12^ (Fig. 1). There was no correlation between data belonging to Caucasian population and our data (r = −0.041, p = 0.768), or between Asian populations and our data (r = 0.152, p = 0.302). In addition, the allele frequencies of RA-associated SNPs varied significantly among different ethnic groups (Fig. 2, Supplementary Fig. 2). The results of allele frequencies were concordant between our study (healthy controls *vs*. RA cases, p-value < 10^−15^ and r = 0.98) and ChileGenomico dataset (healthy controls *vs*. ChileGenomico, p-value < 10^−15^ and r = 0.96). However, the allele frequency in European, East Asian, Aymara and Mapuche samples showed variability compared to our cohort (r ≤ 0.70).

The sliding window test revealed several SNP blocks that were associated with RA (Table 2). The p values for the strongest sliding window (ranging from p = 9.82 × 10^−3^ to 2.04 × 10^−3^) were associated with regions around *STAT4* gene. In addition to the sliding window test, we also performed case-control studies based on linkage disequilibrium (LD) haplotype block reconstruction, not revealing associations between SNPs and RA. Detailed haplotype block information and the LD plot around the *STAT4* gene are shown in Supplementary Fig. 3.

**Table 2:** Association analyses of sliding windows of 4–19 single-nucleotide polymorphisms within STAT4 (only p values < 10^−2^ are shown), using the chi-square test in Plink software (15).

Gene	Markers	Pvalue
*STAT4*	rs3024903|rs3024896|rs4853540|rs16833220	3,94 × 10^−3^
	rs11889341|rs12990918|rs6434435|rs10931480	9,82 × 10^−3^
	rs3024903|rs3024896|rs4853540|rs16833220|rs11893432	6,26 × 10^−3^
	rs3024903|rs3024896|rs4853540|rs16833220|rs11893432|rs3024861	9,59 × 10^−3^
	rs12990918|rs6434435|rs10931480|rs10931481|rs7574865|rs6752770|rs1551440|rs11685878|rs4853546	8,48 × 10^-3^
	rs12990918|rs6434435|rs10931480|rs10931481|rs7574865|rs6752770|rs1551440|rs11685878|rs4853546|rs7574070	3,34 × 10^−3^
	rs12990918|rs6434435|rs10931480|rs10931481|rs7574865|rs6752770|rs1551440|rs11685878|rs4853546|rs7574070|rs12327969	2,04 × 10^−3^
	rs2459611|rs11889341|rs12990918|rs6434435|rs10931480|rs10931481|rs7574865|rs6752770|rs1551440|rs11685878|rs4853546|rs7574070	9,11 × 10^−3^
	rs3024896|rs4853540|rs16833220|rs11893432|rs3024861|rs1517352|rs13426947|rs2459611|rs11889341|rs12990918|rs6434435|rs10931480|rs10931481|rs7574865|rs6752770	7,79 × 10^−3^
	rs3024903|rs3024896|rs4853540|rs16833220|rs11893432|rs3024861|rs1517352|rs13426947|rs2459611|rs11889341|rs12990918|rs6434435|rs10931480|rs10931481|rs7574865|rs6752770|rs1551440	4,32 × 10^−3^
	rs4853540|rs16833220|rs11893432|rs3024861|rs1517352|rs13426947|rs2459611|rs11889341|rs12990918|rs6434435|rs10931480|rs10931481|rs7574865|rs6752770|rs1551440|rs11685878|rs4853546|rs7574070	5,77 × 10^−3^
	rs4853540|rs16833220|rs11893432|rs3024861|rs1517352|rs13426947|rs2459611|rs11889341|rs12990918|`rs6434435|rs10931480|rs10931481|rs7574865|rs6752770|rs1551440|rs11685878|rs4853546|rs7574070|rs12327969	5,88 × 10^−3^

## Discussion

The present study aimed to investigate the association of SNPs markers in candidate genes and RA in the Chilean population. Our main finding was a little replication of previously reported genetic associations with RA. Indeed, only 2% of know RA *loci* from GWAS studies in populations of European or Asian origin were significantly associated in our LA population, and just 11% showed a suggestive association. This was unexpected because SNPs in well-known RA *loci* were tested, such as *PADI4*, *PTPN22*, *STAT4*, *CTLA4*, *TNFAIP3*, and *CCR6* -none of which replicated. There are a number of reasons why previously GWAS-significant findings might not replicate in independent cohorts, as reviewed by Kraft *et al*.^13^. The small sample size of our study may be responsible for the modest number of SNPs that showed associations validated in our participants. Sample sizes larger than the one used here are needed to reach high confidence levels and strong statistical power. In this regard, the low prevalence of the disease restricted the number of patients that we were able to recruit for our study. A long-term effort to progressively collect numerous patients’ samples from biobanks might allow to perform more powered genetic studies and to test for generalizability of genetic associations. Similarly, we believe that the small sample size is a main reason for the lack of differences we found between endophenotypes. Our study did not reach statistical power for one-third of the SNPs analyzed, which might provide a possible explanation, at least in part, for the lack of replication of the results in the Chilean population. However, if lack of power was the only explanation, it is expected that, overall, the OR values would follow the same trend in Chilean patients as in other populations. However, ORs in Chile show absolutely no correlation with estimates from studies with Europeans and only a very week positive association with Asians (Fig. 1). This suggests that genetic divergence between populations at these loci may be one of the reasons of the lack generalization of SNP associations.

Differences in LD patterns between populations may preclude replication of association, which can be caused by multiple factors such as different demographic history including population-specific bottlenecks, genetic drift, selection, and recent admixture, among others^14^. Large diversity in LD among populations from different continents, including the Americas, is well documented^15^. Furthermore, RA is a trait associated with loci responsible for the immune response, which in turn is highly associated with local adaptations and disease resistance. In support for the above interpretation of our results, although we did not find any significant SNP-wise association of *STAT4* with RA, we did find association for this locus when testing haplotypes instead of genotypes. Using the sliding window test revealed several haplotype associations with RA, suggesting the possible existence of untested (potentially functional) genetic variation within *STAT4* in the Chilean population, a result that other studies with different populations might had failed to detect or might had not shown the strongest signal. Further investigations are required to confirm these findings. The strongest association was observed for the SNP rs2451258 located upstream of the T-cell activation RhoGTPase activating protein (*TAGAP*) gene, although the p-value was >0.05 after Bonferroni correction for multiple testing. This variant is not within any protein-coding sequence or disrupted a non-coding functional motif, but *TAGAP* would be a promising biological candidate gene^12^. *TAGAP* gene encodes a member of the Rho GTPase-activator protein superfamily, but little is known about their role in the immune system. Additional investigations, with higher of variants in the region are required to confirm this hypothesis.

Polygenic risk scores could be the next great stride in genomic medicine, which is generating a considerable debate regarding their use in complex phenotypes^16^. Recently, Khera *et al*. proposed that it is time to contemplate the incorporation of polygenic risk prediction in clinical care^17^, projecting these scores across a wide variety of diseases. The risk scores have been generated and tested mainly in individuals of primarily European ancestry. In the present study, significant values of the previously detected SNP-wise associations were moderate and a better generalizability was found when testing association between phenotype and haplotypes rather than SNPs. Moreover, allele frequency vary between populations of different ancestries. These results suggest the existence of genomic patterns in Chilean, and probably other LA populations, that differentiate them from Europeans with regard to *loci* that are relevant for RA. This can be caused by different demographic histories (e.g., past population bottlenecks and migration events, or ancestries^18–20^). Haplotype-based associations may capture the interacting effects among two or more potential causal variants within certain genomic region, which single-variants approach cannot detect. Therefore, haplotype-based approaches show a greater power to map susceptibility genes in complex traits than single-marker methods^21,22^. These results support the need for GWAS in LA populations, including Chileans, to discover potentially novel loci accounting for genetic risk for RA, to investigate the contribution of genetic ancestry, and to improve performance of polygenic prediction models in these populations.

## Methods

### Study participants

A total of 1.340 individuals were studied as two distinct cohorts. Cohort 1 comprised 313 patients with RA and 487 healthy control subjects; cohort 2 included 250 RA patients and 290 healthy controls. The patients with RA were diagnosed following the 2010 American College of Rheumatology/European League Against Rheumatism (ACR/EULAR) classification criteria^23^. The study was approved by the Ethical Committee of the “Servicio de Salud del Maule” (registration number 04/2014), Chile; and all individuals gave their written informed consent prior to enrolling in the study. All methods were performed in accordance with the relevant guidelines and regulations.

### SNP selection and genotyping

A total of 128 SNPs from 73 genes were chosen for genotyping from previous GWAS in populations of diverse ethnic background^7,11^. Supplementary Table 3 shows SNPs elected for our analysis. Some of them were selected as haplotype-tag-SNPs (ht-SNPs) based on LD patterns located within our candidate genes (*PADI4*, *PTPN22*, *STAT4*, *CTLA4*, *TNFAIP3* and *CCR6*) and using the HapMap dataset^24^. Haplotype tagging (Ht)-SNPs were selected using the Tagger tool of Haploview^25^, under the following criteria: minor allele frequency ≥0.01 and r^2^ > 0.8, and based on the HapMap populations (CEU, CEU + TSI and MEX). Some of the identified associations were validated by genotyping 23 SNPs in the cohort 2. The SNPs were genotyped using the OpenArray®™ *TaqMan* platform (Applied Biosystems Inc.) in the test (Cohort 1) and replication (Cohort 2) samples. The genotyping assays were performed at the Pfizer-University of Granada-Junta de Andalucía Centre for Genomics and Oncological Research (GENYO) (Cohort 1), Granada, Spain; and at the Centro Nacional de Genotipado (Cohort 2), at the Santiago de Compostela node, Spain.

### Genotyping data from reference populations

In order to assess ethnic differences in allelic frequencies for the SNPs evaluated in this work, we obtained genotypes for 108 AFR, 99 EUR, and 103 EAS unrelated individuals from the 1000 Genomes Project Phase 3 dataset (http://www.1000genomes.org). For Amerindian ancestry, we obtained genotypes for 85 individuals of Aymara ancestry (AYM), 54 individuals of Mapuche ancestry (MAP), and 348 of Chilean ancestry (CLG) from the ChileGenomico Project (http://chilegenomico.med.uchile.cl). AYM, MAP, and CLG individuals were genotyped using the Axion LAT1 Array (Affymetrix, Inc., Santa Clara, California, U.S.) and imputed using the 1000 Genomes Project phase 3^26^.

### Statistical analysis

Power calculations were done with the GAS Power Calculator tool (http://csg.sph.umich.edu) assuming a multiplicative model, with OR = 1.5, a significance level of 0.05 and an RA prevalence of 0.5%. Only SNPs that met the quality criteria of a minor allele frequency (MAF) > 0.01, missingness < 0.1, and/or HWE P > 0.001 were considered for inclusion in the association analyses (Supplementary Table 3). Allele frequencies were compared between RA patients and control populations by chi-square test, and OR with 95% confidence intervals (95% CI) were calculated using PLINK software (v1.07)^27^. Haplotype analysis was performed using Haploview software (v4.2)^25^. In addition, haplotypes based on 1-bp sliding windows of 2 to 21 SNPs each were also constructed. Association analyses were done with the chi-square test using PLINK. Pearson’s correlations and linear regression were used to evaluate differences between genetic background. The LocusZoom web-based resource was used to generate plots of association results by genomic region^28^.

## Supplementary information

Supplementary information.

## References

[1] Tobón GJ, Youinou P, Saraux A (2010). The environment, geo-epidemiology, and autoimmune disease: Rheumatoid arthritis. Journal of Autoimmunity.

[2] Spindler A (2002). Prevalence of rheumatoid arthritis in Tucuman, Argentina. J. Rheumatol.

[3] Rodrigues Senna É (2004). Prevalence of Rheumatic Diseases in Brazil: A Study Using the COPCORD Approach. Journal of Rheumatology.

[4] Bennett K (1997). Community screening for rheumatic disorder: Cross cultural adaptation and screening characteristics of the COPCORD core questionnaire in Brazil, Chile, and Mexico. Journal of Rheumatology.

[5] MacGregor AJ (2000). Characterizing the quantitative genetic contribution to rheumatoid arthritis using data from twins. Arthritis & Rheumatism.

[6] Gregersen PK, Silver J, Winchester RJ (1987). The shared epitope hypothesis. an approach to understanding the molecular genetics of susceptibility to rheumatoid arthritis. Arthritis & Rheumatism.

[7] Okada Y (2013). Genetics of rheumatoid arthritis contributes to biology and drug discovery. Nature.

[8] Terao C, Raychaudhuri S, Gregersen PK (2016). Recent Advances in Defining the Genetic Basis of Rheumatoid Arthritis. Annual Review of Genomics and Human Genetics.

[9] Yamamoto K, Okada Y, Suzuki A, Kochi Y (2015). Genetics of rheumatoid arthritis in Asia—present and future. Nature Reviews Rheumatology.

[10] Novembre J, Ramachandran S (2011). Perspectives on human population structure at the cusp of the sequencing era. Annu. Rev. Genomics Hum. Genet.

[11] Herráez DL (2013). Rheumatoid arthritis in latin americans enriched for amerindian ancestry is associated with loci in chromosomes 1, 12, and 13, and the HLA Class II region. Arthritis and Rheumatism.

[12] Okada Y (2013). Genetics of rheumatoid arthritis contributes to biology and drug discovery. Nature.

[13] Kraft P, Zeggini E, Ioannidis J (2010). Replication in genome-wide association studies. Stat. Sci..

[14] Slatkin M (1994). Linkage disequilibrium in growing and stable populations. Genetics.

[15] Conrad DF (2006). A worldwide survey of haplotype variation and linkage disequilibrium in the human genome. Nature Genetics.

[16] GWAS to the people. *Nature Medicine* **1483** (2018). 10.1038/s41591-018-0231-3 30297896

[17] Khera AV (2018). Genome-wide polygenic scores for common diseases identify individuals with risk equivalent to monogenic mutations. Nature genetics.

[18] Wang S (2007). Genetic variation and population structure in Native Americans. PLoS Genetics.

[19] Moreno-Estrada A (2014). The genetics of Mexico recapitulates Native American substructure and affects biomedical traits. Science.

[20] Homburger, J. R. *et al*. Genomic Insights into the Ancestry and Demographic History of South America. *Plos Genetics* **11** (2015). 10.1371/journal.pgen.1005602 PMC4670080 26636962

[21] Liu, N., Zhang, K. & Zhao, H. Haplotype-Association Analysis. *Advances in Genetics*, 335–405 (2008). 10.1016/S0065-2660(07)00414-2 18358327

[22] Hsieh AR, Hsiao CL, Chang SW, Wang HM, Fann CSJ (2011). On the use of multifactor dimensionality reduction (MDR) and classification and regression tree (CART) to identify haplotype-haplotype interactions in genetic studies. Genomics.

[23] Aletaha D (2010). 2010 Rheumatoid arthritis classification criteria: An American College of Rheumatology/European League Against Rheumatism collaborative initiative. Arthritis and Rheumatism.

[24] Frazer KA (2007). A second generation human haplotype map of over 3.1 million SNPs. Nature.

[25] Barrett JC, Fry B, Maller J, Daly MJ (2005). Haploview: Analysis and visualization of LD and haplotype maps. Bioinformatics.

[26] Auton A (2015). A global reference for human genetic variation. Nature.

[27] Purcell S (2007). PLINK: A tool set for whole-genome association and population-based linkage analyses. American Journal of Human Genetics.

[28] Pruim RJ (2011). LocusZoom: Regional visualization of genome-wide association scan results. In Bioinformatics.

