# Supplementary material for: Association analysis in a Latin American population revealed ethnic differences in rheumatoid arthritis-associated SNPs in Caucasian and Asian populations
Source: Sci Rep. 2020 May 12;10:7879. doi: 10.1038/s41598-020-64659-0 (PMC7217883; doi:10.1038/s41598-020-64659-0)
Supplement: Supplementary file 1 — Supplementary information. [file 41598_2020_64659_MOESM1_ESM.doc]

**SUPPLEMENTARY INFORMATION**

**Title:** Association analysis in a Latin American population revealed ethnic differences in rheumatoid arthritis-associated SNPs in Caucasian and Asian populations

**Authors:**

Castro-Santos P^1,2^, Verdugo RA^3,4^, Alonso-Arias R^1^, Gutiérrez MA^5^, Suazo J^6^, Aguillón JC^7^, Olloquequi J^1^, Pinochet C^8^, Lucia A^9^, Quiñones LA^≠10^, Díaz-Peña R^≠1^.

**Affiliations:**

^1^Facultad de Ciencias de la Salud, Universidad Autónoma de Chile, Talca, Chile.

^2^Inmunología, Centro de Investigaciones Biomédicas (CINBIO), Universidad de Vigo, Vigo, Spain.

^3^Programa de Genética Humana, ICBM, Facultad de Medicina, Universidad de Chile, Santiago, Chile.

^4^Departamento de Oncología Básico Clínica, Facultad de Medicina, Universidad de Chile, Santiago, Chile.

^5^Departamento de Inmunología Clínica y Reumatología, Facultad de Medicina, Pontificia Universidad Católica de Chile, Santiago, Chile.

^6^Instituto de Investigación en Ciencias Odontológicas, Facultad de Odontología, Universidad de Chile, Santiago, Chile.

^7^Immune Regulation and Tolerance Research Group, Programa de Inmunología, ICBM, Facultad de Medicina, Universidad de Chile, Santiago, Chile.

^8^Hospital Regional de Talca, Talca, Chile.

^9^Universidad Europea de Madrid (Faculty of Sports Sciences) and Research Institute Hospital 12 de Octubre (‘i+12’), Madrid, Spain.

^10^Laboratorio de Carcinogénesis Química y Farmacogenética, Programa de Farmacología Molecular y Clínica, ICBM, Facultad de Medicina, Universidad de Chile, Santiago, Chile.

**Supplementary Table 1**. Selected genome-wide association studies (GWAS) and fine-mapping studies on rheumatoid arthritis (RA).

| **Year** | **Initial Sample**  **Size** | **Replication Sample**  **Size** | **nº SNPs** | **Ref** |
| --- | --- | --- | --- | --- |
| 2007 | 1,860 EU ancestry cases  2,938 EU ancestry controls | NA | ~500.000 | (1) |
| 2007 | 1,522 EU ancestry cases  1,850 EU ancestry controls | 1,053 EU ancestry cases  1,858 EU ancestry controls | ~300.000 | (2) |
| 2008 | 400 EU ancestry cases  400 EU ancestry controls | 410 EU ancestry cases  394 EU ancestry controls | ~300.000 | (3) |
| 2008 | 3,393 EU ancestry cases  12,460 EU ancestry controls | 3,929 EU ancestry cases  5,807 EU ancestry controls | Meta-Analysis | (4) |
| 2010 | 2,303 JP ancestry cases  3,380 JP ancestry controls | 4,768 JP ancestry cases  17,359 JP ancestry controls | 550.000 | (5) |
| 2010 | 5,539 EU ancestry cases  20,169 EU ancestry controls | 6,768 EU ancestry cases  8,806 EU ancestry controls | ∼900 000 | (6) |
| 2011 | 1,147 EU ancestry ACPA (+) cases  774 EU ancestry ACPA (-) cases  1,079 EU ancestry controls | 887 EU ancestry ACPA (+) cases  1,218 EU ancestry controls | >1.5 x10^6^ | (7) |
| 2011 | 801 KR ancestry cases  757 KR ancestry controls | 718 KR ancestry cases  719 KR ancestry controls | ~450.000 | (8) |
| 2011 | 1,247 JP ancestry cases  1,486 JP ancestry controls | 4,168 JP ancestry cases  5,003 JP ancestry controls | ~500.000 | (9) |
| 2011 | 100 KR ancestry cases  600 KR ancestry controls | 578 KR ancestry cases  711 KR ancestry controls | ~300.000 | (10) |
| 2013 | 706 NI ancestry cases  761 NI ancestry controls | 927 NI ancestry cases  1,148 NI ancestry controls | >450.000 | (11) |
| 2014 | 3,034 EU ancestry cases  5,271 EU ancestry controls | 4,726 EU ancestry cases  2,625 EU ancestry controls | <2.000.000 | (12) |
| 2014 | up to 14,361 EU ancestry cases  up to 42,923 EU ancestry controls  up to 4,873 EA ancestry cases  up to 17,642 EA ancestry controls | up to 3,775 EU ancestry cases  up to 5,801 EU ancestry controls  up to 6,871 EA ancestry cases  up to 6,392 EA ancestry controls | ~10x10^6^ | (13) |
| 2014 | 433 CH ancestry ACPA (+) cases  519 CH ancestry ACPA (-) cases  943 CH ancestry controls | 1,032 CH ancestry ACPA (+) cases  1,100 CH ancestry ACPA (-) cases  2,553 CH ancestry controls | ∼900 000 | (14) |
| 2015 | 1,148 EU ancestry anti-CCP (-) cases  6,008 EU ancestry controls | 774 EU ancestry anti-CCP (-) cases  1,079 EU ancestry controls | ~450.000 | (15) |
| 2017 | 511 AS ancestry cases  352 AS ancestry controls | 283 AS ancestry cases  221 AS ancestry controls | >7x10^6^ | (16) |
| 2017 | 3,323 EU ancestry cases  15,785 EU ancestry controls | NA | ~100.000 | (17) |
| 2019 | 868 EU ancestry cases  1,194 EU ancestry controls | NA | ~500.000 | (18) |
| 2019 | 916 AA ancestry cases  1,392 AA ancestry controls | *19,234 EU ancestry cases  *61,654 EU ancestry controls  *4,873 EAS ancestry cases  *17,641 EAS ancestry controls | >8x10^6^ | (19) |

*previously published (13).

ACPA=anti-citrullinated protein antibodies; SNP=single nucleotide polymorphism; AS=Arab States; CH=Chinese Han; EU=European; JP=Japanese; KR=Korean; NI=North India; AA=African-Americans; EAS=East Asian.

**Supplementary Table 2**. Characteristics of study participants.

|  | Cohort 1 | Cohort 2 |
| --- | --- | --- |
| **Demographic characteristics** |  |  |
| Women, n (%) | 265 (85%) | 202 (81%) |
| Men, n (%) | 48 (15%) | 48 (19%) |
| Age, median (range) | 48 (20-75) | 50 (17-76) |
| **Clinical Parameters** |  |  |
| Disease duration, median (range) | 8 (1-42) | 8 (1-37) |
| Age of onset | 40 (15-67) | 37 (10-69) |
| ACPA positivity, n ^a^ | 112 | 52 |
| RF, n ^b^ | 170 | 94 |

^a^ A total of 164 patients were positive for ACPA. The remainder were either negative or had no available data regarding ACPA status.

^b^ A total of 264 patients were positive for RF. The remainder were either negative or had no available data regarding RF status.

ACPA=anti-citrullinated protein antibodies; RF=rheumatic factor.

**Supplementary Table 3**. Association results for the 128 SNPs included in the study (cohort 1).

| ID | SNP | CHR | Position | Gene | A1 | MAF  RA cases | MAF  controls | Quality Control | P value | OR | L95 | U95 |
| --- | --- | --- | --- | --- | --- | --- | --- | --- | --- | --- | --- | --- |
| 1 | rs2843401 | 1 | 2528133 | MMEL1 | T | 0.41 | 0.46 | pass | 0.05809 | 0.816 | 0.6611 | 1.007 |
| 2 | rs2477137 | 1 | 17630605 | PADI4 | T | 0.18 | 0.21 | pass | 0.2247 | 0.8476 | 0.6489 | 1.107 |
| 3 | rs11800688 | 1 | 17631652 | PADI4 | T | 0.24 | 0.21 | HWE p < 0.001 | 0.3007 | 1.149 | 0.8834 | 1.493 |
| 4 | rs1886303 | 1 | 17632382 | PADI4 | T | 0.40 | 0.43 | pass | 0.3868 | 0.9107 | 0.7367 | 1.126 |
| 5 | rs2501795 | 1 | 17633499 | PADI4 | G | 0.11 | 0.09 | pass | 0.2688 | 1.219 | 0.8577 | 1.733 |
| 6 | rs2477134 | 1 | 17633572 | PADI4 | G | 0.37 | 0.31 | pass | 0.01563 | 1.315 | 1.053 | 1.642 |
| 7 | rs1886302 | 1 | 17635396 | PADI4 | C | 0.46 | 0.45 | pass | 0.5642 | 1.063 | 0.8631 | 1.31 |
| 8 | rs1748041 | 1 | 17655407 | PADI4 | C | 0.42 | 0.39 | pass | 0.2628 | 1.129 | 0.9127 | 1.397 |
| 9 | rs11203367 | 1 | 17657616 | PADI4 | T | 0.47 | 0.44 | pass | 0.2789 | 1.123 | 0.9105 | 1.384 |
| 10 | rs2301888 | 1 | 17672730 | PADI4 | A | 0.43 | 0.43 | pass | 0.8965 | 0.9861 | 0.7988 | 1.217 |
| 11 | rs2240336 | 1 | 17674402 | PADI4 | T | 0.46 | 0.46 | pass | 0.9336 | 1.009 | 0.8197 | 1.242 |
| 12 | rs2240335 | 1 | 17674537 | PADI4 | A | 0.42 | 0.42 | pass | 0.9725 | 0.9962 | 0.8032 | 1.236 |
| 13 | rs1635567 | 1 | 17683041 | PADI4 | C | 0.33 | 0.41 | pass | 0.004623 | 0.7296 | 0.5864 | 0.9078 |
| 14 | rs1635561 | 1 | 17687027 | PADI4 | T | 0.14 | 0.15 | pass | 0.5953 | 0.9212 | 0.6806 | 1.247 |
| 15 | rs28411352 | 1 | 38278579 | MTF1-INPP5B | T | 0.25 | 0.23 | pass | 0.3432 | 1.124 | 0.8828 | 1.43 |
| 16 | rs883220 | 1 | 38616871 | POU3F1 | A | 0.11 | 0.11 | pass | 0.6765 | 0.9304 | 0.663 | 1.306 |
| 17 | rs3811021 | 1 | 114356663 | PTPN22 | G | 0.12 | 0.13 | pass | 0.7576 | 0.9502 | 0.6871 | 1.314 |
| 18 | rs1217413 | 1 | 114357750 | PTPN22 | G | 0.44 | 0.41 | pass | 0.3012 | 1.119 | 0.9041 | 1.385 |
| 19 | rs2797416 | 1 | 114369259 | PTPN22 | C | 0.42 | 0.43 | pass | 0.7713 | 0.9674 | 0.7737 | 1.21 |
| 20 | rs2476601 | 1 | 114377568 | PTPN22 | A | 0.08 | 0.07 | pass | 0.3523 | 1.211 | 0.8086 | 1.814 |
| 21 | rs3789609 | 1 | 114397799 | PTPN22 | T | 0.22 | 0.22 | pass | 0.9918 | 1.001 | 0.7769 | 1.291 |
| 22 | rs2488458 | 1 | 114406451 | PTPN22 | T | 0.46 | 0.44 | pass | 0.5221 | 1.071 | 0.868 | 1.321 |
| 23 | rs1217414 | 1 | 114412667 | PTPN22 | A | 0.18 | 0.21 | pass | 0.1558 | 0.8221 | 0.6271 | 1.078 |
| 24 | rs624988 | 1 | 117263790 | CD2 | T | 0.46 | 0.46 | pass | 0.9611 | 1.005 | 0.8158 | 1.239 |
| 25 | rs2228145 | 1 | 154426970 | IL6R | A | 0.42 | 0.38 | pass | 0.09257 | 1.204 | 0.9697 | 1.494 |
| 26 | rs72717009 | 1 | 161405053 | FCGR2A | A | 0.22 | 0.18 | pass | 0.1361 | 1.223 | 0.9383 | 1.595 |
| 27 | rs2105325 | 1 | 173349725 | LOC100506023 | A | 0.36 | 0.37 | pass | 0.7082 | 0.9579 | 0.7646 | 1.2 |
| 28 | rs2014863 | 1 | 198809601 | PTPRC | G | 0.23 | 0.22 | pass | 0.6313 | 1.063 | 0.8277 | 1.366 |
| 29 | rs10175798 | 2 | 30449594 | LBH | G | 0.33 | 0.37 | pass | 0.06833 | 0.8153 | 0.6545 | 1.016 |
| 30 | rs34695944 | 2 | 61124850 | REL | C | 0.24 | 0.24 | HWE p < 0.001 | 0.8829 | 0.9816 | 0.7668 | 1.257 |
| 31 | rs1858037 | 2 | 65598300 | SPRED2 | T | 0.38 | 0.34 | HWE p < 0.001 | 0.1834 | 1.162 | 0.9313 | 1.45 |
| 32 | rs9653442 | 2 | 100825367 | AFF3 | T | 0.42 | 0.45 | pass | 0.2088 | 0.8724 | 0.7051 | 1.079 |
| 33 | rs6732565 | 2 | 111607832 | ACOXL | A | 0.45 | 0.46 | HWE p < 0.001 | 0.6519 | 0.9527 | 0.7717 | 1.176 |
| 34 | rs3024903 | 2 | 191895607 | STAT4 | T | 0.15 | 0.11 | pass | 0.03052 | 1.399 | 1.031 | 1.898 |
| 35 | rs3024896 | 2 | 191896716 | STAT4 | T | 0.15 | 0.13 | pass | 0.2681 | 1.189 | 0.8751 | 1.615 |
| 36 | rs4853540 | 2 | 191917317 | STAT4 | T | 0.24 | 0.26 | pass | 0.4942 | 0.9187 | 0.7203 | 1.172 |
| 37 | rs16833220 | 2 | 191917344 | STAT4 | G | 0.13 | 0.10 | pass | 0.1751 | 1.252 | 0.9044 | 1.733 |
| 38 | rs11893432 | 2 | 191921874 | STAT4 | G | 0.47 | 0.43 | pass | 0.1831 | 1.155 | 0.9341 | 1.428 |
| 39 | rs3024861 | 2 | 191924606 | STAT4 | A | 0.48 | 0.49 | pass | 0.7261 | 0.9628 | 0.7787 | 1.19 |
| 40 | rs1517352 | 2 | 191931464 | STAT4 | C | 0.36 | 0.39 | pass | 0.2598 | 0.8818 | 0.7084 | 1.098 |
| 41 | rs13426947 | 2 | 191933254 | STAT4 | T | 0.40 | 0.36 | pass | 0.1571 | 1.168 | 0.9418 | 1.449 |
| 42 | rs2459611 | 2 | 191939187 | STAT4 | C | 0.11 | 0.09 | pass | 0.1579 | 1.285 | 0.9067 | 1.821 |
| 43 | rs11889341 | 2 | 191943742 | STAT4 | T | 0.42 | 0.38 | pass | 0.07397 | 1.217 | 0.981 | 1.511 |
| 44 | rs12990918 | 2 | 191953389 | STAT4 | T | 0.08 | 0.06 | pass | 0.3655 | 1.207 | 0.8027 | 1.815 |
| 45 | rs6434435 | 2 | 191953864 | STAT4 | A | 0.13 | 0.12 | pass | 0.4475 | 1.131 | 0.8231 | 1.554 |
| 46 | rs10931480 | 2 | 191954047 | STAT4 | G | 0.16 | 0.14 | pass | 0.3252 | 1.159 | 0.8638 | 1.554 |
| 47 | rs10931481 | 2 | 191954852 | STAT4 | A | 0.44 | 0.47 | pass | 0.2378 | 0.8777 | 0.7067 | 1.09 |
| 48 | rs7574865 | 2 | 191964633 | STAT4 | T | 0.44 | 0.40 | pass | 0.1544 | 1.166 | 0.9439 | 1.44 |
| 49 | rs6752770 | 2 | 191973563 | STAT4 | G | 0.27 | 0.29 | pass | 0.4347 | 0.9095 | 0.7167 | 1.154 |
| 50 | rs1551440 | 2 | 191996006 | STAT4 | G | 0.29 | 0.27 | pass | 0.4848 | 1.086 | 0.8619 | 1.368 |
| 51 | rs11685878 | 2 | 192009455 | STAT4 | T | 0.33 | 0.34 | pass | 0.6711 | 0.9513 | 0.7554 | 1.198 |
| 52 | rs4853546 | 2 | 192009652 | STAT4 | A | 0.27 | 0.28 | pass | 0.6358 | 0.9427 | 0.7384 | 1.203 |
| 53 | rs7574070 | 2 | 192010488 | STAT4 | A | 0.32 | 0.34 | pass | 0.5967 | 0.9411 | 0.7515 | 1.178 |
| 54 | rs12327969 | 2 | 192010771 | STAT4 | C | 0.21 | 0.21 | pass | 0.883 | 0.9808 | 0.7577 | 1.27 |
| 55 | rs1980422 | 2 | 204610396 | CD28 | C | 0.21 | 0.20 | pass | 0.6388 | 1.063 | 0.8233 | 1.373 |
| 56 | rs5742909 | 2 | 204732347 | CTLA4 | T | 0.10 | 0.09 | pass | 0.812 | 1.044 | 0.7321 | 1.489 |
| 57 | rs231775 | 2 | 204732714 | CTLA4 | G | 0.36 | 0.33 | pass | 0.2482 | 1.138 | 0.9137 | 1.418 |
| 58 | rs3087243 | 2 | 204738919 | CTLA4 | A | 0.47 | 0.50 | pass | 0.2634 | 0.8854 | 0.7154 | 1.096 |
| 59 | rs11571302 | 2 | 204742934 | CTLA4 | G | 0.52 | 0.47 | pass | 0.08813 | 1.199 | 0.9732 | 1.478 |
| 60 | rs4452313 | 3 | 17047032 | PLCL2 | T | 0.30 | 0.27 | pass | 0.2872 | 1.143 | 0.8936 | 1.462 |
| 61 | rs3806624 | 3 | 27764623 | EOMES | A | 0.32 | 0.32 | pass | 0.8414 | 1.024 | 0.8131 | 1.289 |
| 62 | rs13142500 | 4 | 10727357 | CLNK | C | - | - | genotyped <90% samples | - | - | - | - |
| 63 | rs11933540 | 4 | 26120001 | C4orf52 | C | 0.21 | 0.20 | pass | 0.5659 | 1.079 | 0.8321 | 1.4 |
| 64 | rs2664035 | 4 | 48220839 | TEC | A | 0.39 | 0.44 | pass | 0.06439 | 0.8161 | 0.6579 | 1.012 |
| 65 | rs7731626 | 5 | 55444683 | ANKRD55 | A | 0.32 | 0.34 | pass | 0.2867 | 0.8824 | 0.7008 | 1.111 |
| 66 | rs2561477 | 5 | 102608924 | C5orf30 | A | 0.20 | 0.19 | pass | 0.5468 | 1.085 | 0.8329 | 1.412 |
| 67 | rs17264332 | 6 | 138005515 | TNFAIP3 | C | 0.38 | 0.34 | pass | 0.09912 | 1.209 | 0.9648 | 1.514 |
| 68 | rs6920220 | 6 | 138006504 | TNFAIP3 | A | 0.15 | 0.12 | pass | 0.2051 | 1.218 | 0.8975 | 1.653 |
| 69 | rs3757173 | 6 | 138190154 | TNFAIP3 | G | 0.11 | 0.09 | pass | 0.3442 | 1.184 | 0.8338 | 1.682 |
| 70 | rs719149 | 6 | 138192745 | TNFAIP3 | A | 0.13 | 0.12 | HWE p < 0.001 | 0.5179 | 1.11 | 0.8087 | 1.524 |
| 71 | rs582757 | 6 | 138197824 | TNFAIP3 | C | 0.26 | 0.25 | HWE p < 0.001 | 0.5338 | 1.081 | 0.8454 | 1.383 |
| 72 | rs610604 | 6 | 138199417 | TNFAIP3 | G | 0.36 | 0.36 | pass | 0.9815 | 0.9974 | 0.8022 | 1.24 |
| 73 | rs2451258 | 6 | 159506600 | TAGAP | C | 0.15 | 0.19 | pass | 0.03788 | 0.7402 | 0.5568 | 0.984 |
| 74 | rs3093023 | 6 | 167534290 | CCR6 | A | 0.29 | 0.31 | pass | 0.5074 | 0.9237 | 0.7304 | 1.168 |
| 75 | rs1855025 | 6 | 167537594 | CCR6 | A | 0.36 | 0.39 | pass | 0.3815 | 0.9068 | 0.7283 | 1.129 |
| 76 | rs1571878 | 6 | 167540842 | CCR6 | C | 0.31 | 0.32 | pass | 0.6176 | 0.9432 | 0.7497 | 1.187 |
| 77 | rs3798315 | 6 | 167544709 | CCR6 | T | 0.07 | 0.08 | pass | 0.7432 | 0.9365 | 0.6324 | 1.387 |
| 78 | rs3093012 | 6 | 167548281 | CCR6 | A | 0.33 | 0.36 | pass | 0.1894 | 0.8651 | 0.6966 | 1.074 |
| 79 | rs3093010 | 6 | 167548607 | CCR6 | C | 0.47 | 0.47 | pass | 0.959 | 1.006 | 0.8157 | 1.239 |
| 80 | rs3093009 | 6 | 167549477 | CCR6 | G | 0.15 | 0.14 | pass | 0.5165 | 1.101 | 0.824 | 1.47 |
| 81 | rs3093007 | 6 | 167549775 | CCR6 | C | 0.11 | 0.11 | pass | 0.9079 | 1.02 | 0.7299 | 1.425 |
| 82 | rs3093006 | 6 | 167551452 | CCR6 | A | 0.08 | 0.09 | pass | 0.7091 | 0.9315 | 0.6416 | 1.352 |
| 83 | rs67250450 | 7 | 28174986 | JAZF1 | T | 0.46 | 0.49 | pass | 0.2854 | 0.8883 | 0.7148 | 1.104 |
| 84 | rs4272 | 7 | 92236829 | CDK6 | G | 0.21 | 0.21 | pass | 0.8575 | 1.024 | 0.7924 | 1.323 |
| 85 | rs3778753 | 7 | 128580042 | IRF5 | C | 0.34 | 0.28 | pass | 0.01787 | 1.311 | 1.048 | 1.642 |
| 86 | rs2736337 | 8 | 11341880 | BLK | C | - | - | genotyped <90% samples | - | - | - | - |
| 87 | rs998731 | 8 | 81095395 | TDP52 | C | 0.36 | 0.31 | pass | 0.05147 | 1.249 | 0.9984 | 1.561 |
| 88 | rs678347 | 8 | 102463602 | GRHL2 | A | 0.51 | 0.49 | pass | 0.4174 | 1.092 | 0.8832 | 1.349 |
| 89 | rs1516971 | 8 | 129542100 | PVT1 | C | 0.06 | 0.08 | pass | 0.1632 | 0.7508 | 0.5013 | 1.125 |
| 90 | rs11574914 | 9 | 34710338 | CCL19-CCL21 | A | 0.28 | 0.26 | pass | 0.5545 | 1.074 | 0.8478 | 1.36 |
| 91 | rs10985070 | 9 | 123636121 | TRAF1-C5 | C | 0.36 | 0.35 | pass | 0.7305 | 1.039 | 0.8344 | 1.295 |
| 92 | rs706778 | 10 | 6098949 | IL2RA | T | 0.51 | 0.47 | pass | 0.1519 | 1.165 | 0.9453 | 1.436 |
| 93 | rs947474 | 10 | 6390450 | PRKCQ | G | 0.15 | 0.13 | pass | 0.4282 | 1.129 | 0.8357 | 1.527 |
| 94 | rs2275806 | 10 | 8095340 | GATA3 | A | 0.38 | 0.43 | pass | 0.05166 | 0.8091 | 0.6535 | 1.002 |
| 95 | rs793108 | 10 | 31415106 | ZNF438 | C | 0.38 | 0.40 | pass | 0.4436 | 0.9185 | 0.739 | 1.142 |
| 96 | rs2671692 | 10 | 50097819 | WDFY4 | G | 0.43 | 0.41 | pass | 0.4292 | 1.089 | 0.881 | 1.347 |
| 97 | rs71508903 | 10 | 63779871 | ARID5B | T | 0.20 | 0.19 | pass | 0.6281 | 1.067 | 0.8206 | 1.388 |
| 98 | rs12764378 | 10 | 63800004 | ARID5B | A | 0.14 | 0.15 | pass | 0.7937 | 0.9608 | 0.7117 | 1.297 |
| 99 | rs570676 | 11 | 36492191 | TRAF6 | T | 0.27 | 0.30 | pass | 0.2885 | 0.8827 | 0.7011 | 1.111 |
| 100 | rs595158 | 11 | 60909581 | CD5 | C | 0.30 | 0.28 | pass | 0.3854 | 1.105 | 0.8817 | 1.386 |
| 101 | rs968567 | 11 | 61595564 | FADS1-FADS2-FADS3 | A | 0.07 | 0.10 | pass | 0.07234 | 0.7023 | 0.477 | 1.034 |
| 102 | rs4409785 | 11 | 95311422 | CEP57 | C | 0.16 | 0.12 | pass | 0.02855 | 1.397 | 1.035 | 1.886 |
| 103 | rs4938573 | 11 | 118741842 | DDX6 | C | 0.12 | 0.13 | pass | 0.8733 | 0.9727 | 0.6918 | 1.367 |
| 104 | rs73013527 | 11 | 128496952 | ETS1 | T | 0.36 | 0.41 | pass | 0.07266 | 0.8189 | 0.6584 | 1.019 |
| 105 | rs773125 | 12 | 56394954 | CDK2 | G | 0.31 | 0.33 | pass | 0.33 | 0.8873 | 0.6974 | 1.129 |
| 106 | rs1633360 | 12 | 58108052 | CDK4 | T | - | - | MAF < 0.01 | - | - | - | - |
| 107 | rs10774624 | 12 | 111833788 | SH2B3-PTPN11 | G | 0.31 | 0.30 | pass | 0.6958 | 1.047 | 0.8327 | 1.316 |
| 108 | rs4766764 | 12 | 112493067 | NAA25 | A | 0.13 | 0.16 | pass | 0.1303 | 0.7968 | 0.5935 | 1.07 |
| 109 | rs9603616 | 13 | 40368069 | COG6 | T | 0.21 | 0.24 | pass | 0.2181 | 0.854 | 0.6642 | 1.098 |
| 110 | rs1950897 | 14 | 68760141 | RAD51B | G | 0.21 | 0.25 | pass | 0.07819 | 0.7996 | 0.6233 | 1.026 |
| 111 | rs8043085 | 15 | 38828140 | RASGRP1 | T | 0.31 | 0.30 | pass | 0.8163 | 1.027 | 0.8194 | 1.287 |
| 112 | rs8032939 | 15 | 38834033 | RASGRP1 | C | 0.35 | 0.35 | pass | 0.9408 | 1.008 | 0.8125 | 1.251 |
| 113 | rs8026898 | 15 | 69991417 | TLE3 | A | 0.27 | 0.26 | pass | 0.69 | 1.049 | 0.8299 | 1.325 |
| 114 | rs4780401 | 16 | 11839326 | TNXNDC11 | T | 0.40 | 0.35 | pass | 0.07873 | 1.215 | 0.9778 | 1.509 |
| 115 | rs13330176 | 16 | 86019087 | IRF8 | A | 0.49 | 0.51 | pass | 0.5442 | 0.9368 | 0.7586 | 1.157 |
| 116 | rs72634030 | 17 | 5272580 | C1QBP | A | 0.24 | 0.24 | pass | 0.8161 | 1.03 | 0.8058 | 1.315 |
| 117 | rs1877030 | 17 | 37740161 | MED1 | T | 0.18 | 0.19 | pass | 0.6665 | 0.9428 | 0.7213 | 1.232 |
| 118 | rs12936409 | 17 | 38043649 | IKZF3 | A | 0.46 | 0.42 | pass | 0.1297 | 1.179 | 0.9527 | 1.459 |
| 119 | rs2469434 | 18 | 67544046 | CD226 | C | 0.32 | 0.26 | pass | 0.008587 | 1.355 | 1.08 | 1.701 |
| 120 | rs6032662 | 20 | 44734310 | CD40 | C | 0.21 | 0.18 | pass | 0.09102 | 1.256 | 0.964 | 1.636 |
| 121 | rs4239702 | 20 | 44749251 | CD40 | T | 0.22 | 0.18 | pass | 0.04387 | 1.305 | 1.007 | 1.691 |
| 122 | rs2834512 | 21 | 35911599 | RCAN1 | A | 0.10 | 0.08 | pass | 0.238 | 1.242 | 0.8659 | 1.783 |
| 123 | rs9979383 | 21 | 36715761 | RUNX1 | C | 0.31 | 0.25 | pass | 0.01809 | 1.322 | 1.048 | 1.666 |
| 124 | rs1893592 | 21 | 43855067 | UBASH3A | C | 0.34 | 0.34 | pass | 0.8956 | 1.015 | 0.8141 | 1.265 |
| 125 | rs11089637 | 22 | 21979096 | UBE2L3-YDJC | C | 0.43 | 0.41 | pass | 0.5995 | 1.058 | 0.8567 | 1.307 |
| 126 | rs3218251 | 22 | 37545505 | IL2RB | A | 0.18 | 0.14 | pass | 0.03742 | 1.351 | 1.017 | 1.796 |
| 127 | rs909685 | 22 | 39747671 | SYNGR1 | A | 0.47 | 0.48 | pass | 0.5123 | 0.932 | 0.7549 | 1.151 |
| 128 | rs5987194 | X | 153301467 | IRAK1 | C | 0.44 | 0.44 | HWE p < 0.001 | 0.9855 | 0.998 | 0.8076 | 1.233 |

SNP=Single nucleotide polymorphism; CHR=Chromosome; A1=Minor allele nucleotide; MAF=Minor allele frequency; OR=Odds ratio; L95=Lower bound on confidence interval for odds ratio; U95=Upper bound on confidence interval for odds ratio.


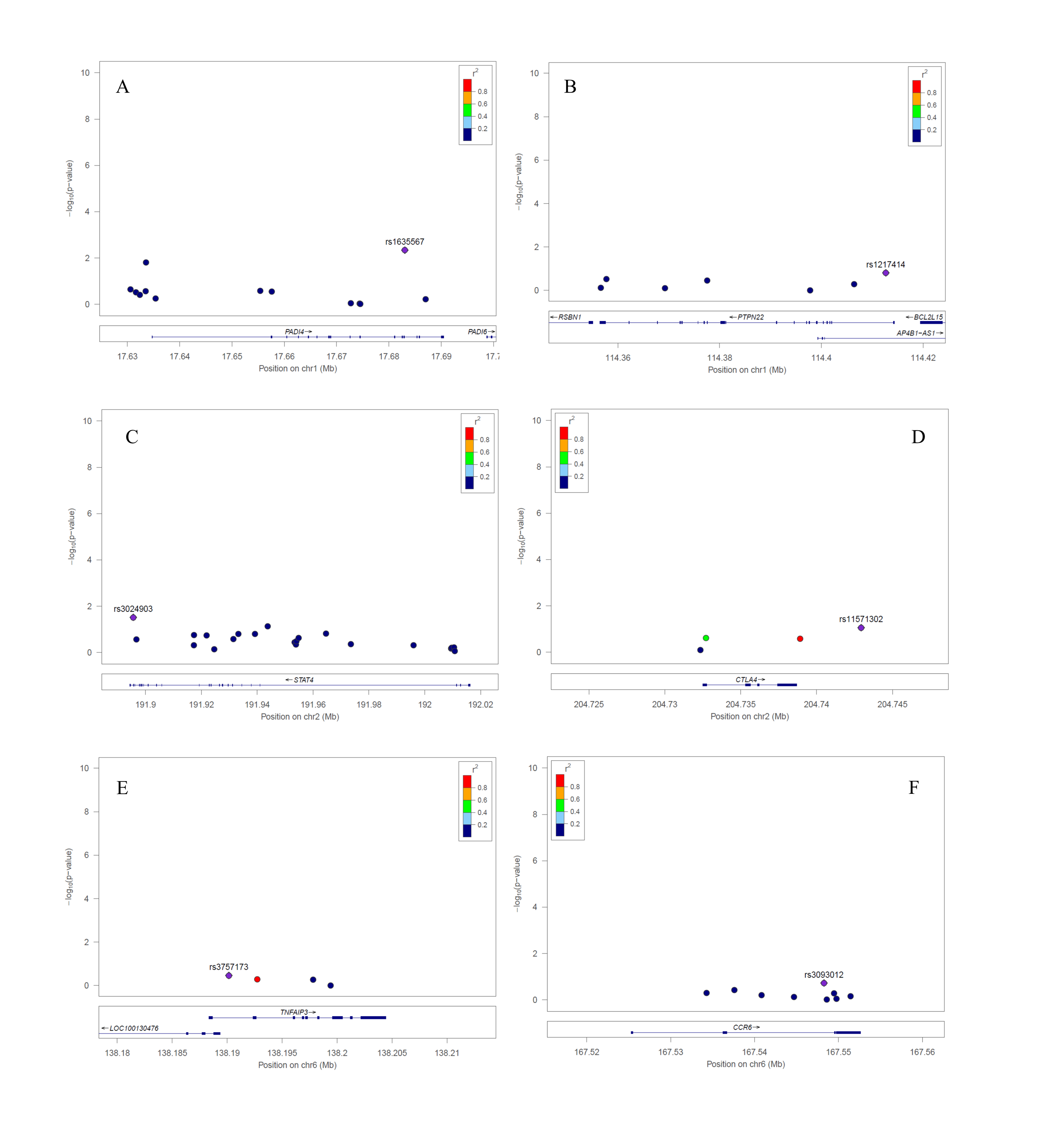
**Supplementary Figure 1.** Regional plot of results of the association between SNPs in (A) *PADI4*, (B) *PTPN22*, (C) *STAT4*, (D) *CTLA4*, (E) *TNFAIP3*, and (F) *CCR6* and risk of RA.

**Supplementary Figure 2.** Correlation between the allele frequency difference for the candidate SNPs in the Chilean population of our study versus European or Asian populations (1000 Genomes Project Phase 3 dataset, http://www.1000genomes.org) (r=0.98, r=0.68 and r=0.65; respectively).

**
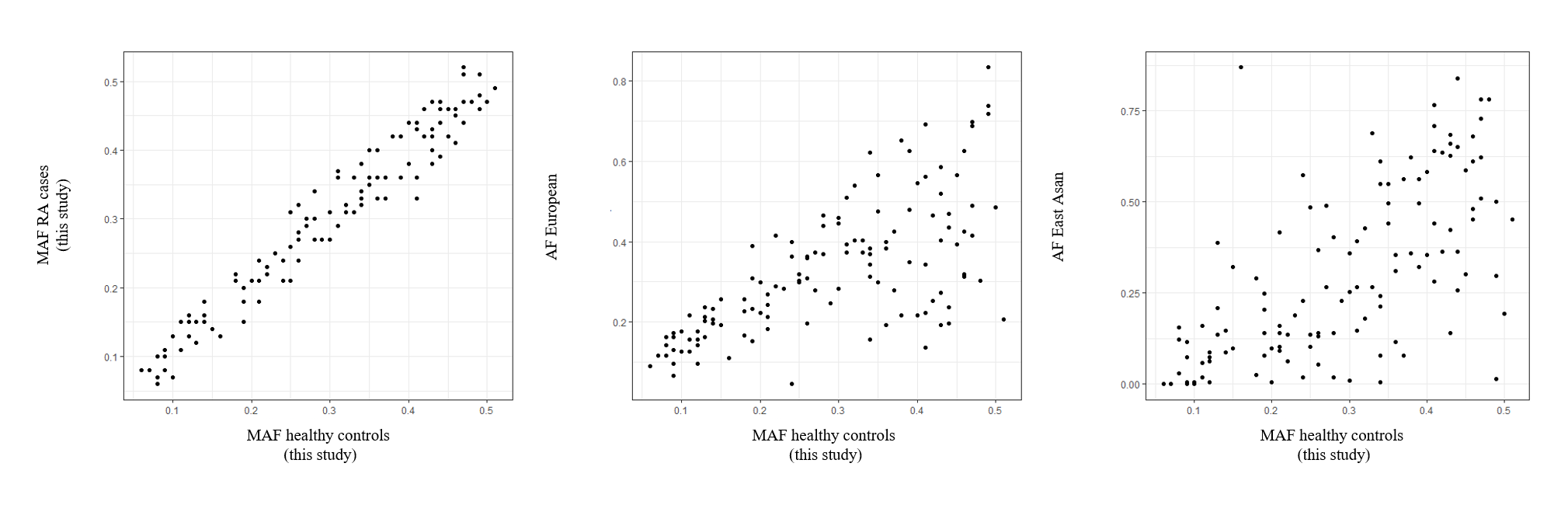
**

MAF=minor allele frequency; AF=allele frequency.

**Supplementary Figure 3**. The bottom panel shows the linkage disequilibrium (LD) among 21 selected SNPs within *STAT4* (squares represent the LD (r^2^) value between every 2 single-nucleotide polymorphisms: the blacker the square, the higher the r^2^ pairwise value). The top panel shows the P value of association between SNPs and RA.


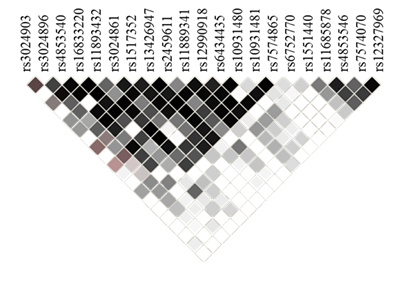


**REFERENCES:**

1. Wellcome T, Case T, Consortium C. Genome-wide association study of 14,000 cases of seven common diseases and 3,000 shared controls. Nature. 2007;447(7145):661–78.

2. Plenge RM, Seielstad M, Padyukov L, et al. TRAF1-C5 as a risk locus for rheumatoid arthritis--a genomewide study. New England Journal of Medicine. 2007;357(12):1199–209.

3. Julia A, Ballina J, Canete JD, et al. Genome-wide association study of rheumatoid arthritis in the Spanish population. Arthritis and Rheumatism. 2008;58:2275–86.

4. Raychaudhuri S, Remmers EF, Lee AT, et al. Common variants at CD40 and other loci confer risk of rheumatoid arthritis. Nature Genetics . 2008;40(10):1216–23.

5. Kochi Y, Okada Y, Suzuki A, et al. A regulatory variant in CCR6 is associated with rheumatoid arthritis susceptibility. Nature Genetics. 2010;42(6):515–9.

6. Stahl EA, Raychaudhuri S, Remmers EF, et al. Genome-wide association study meta-analysis identifies seven new rheumatoid arthritis risk loci. Nature Genetics. 2010;42(6):508–14.

7. Padyukov L, Seielstad M, Ong RTH, et al. A genome-wide association study suggests contrasting associations in. Annals of the rheumatic diseases. 2011;70(2):259–65.

8. Freudenberg J, Lee HS, Han BG, et al. Genome-Wide Association Study of Rheumatoid Arthritis in Koreans: Population-Specific Loci as Well as Overlap with European Susceptibility Loci. Arthritis and Rheumatism. 2011;63(4):884–93.

9. Terao C, Yamada R, Ohmura K, et al. The human AIRE gene at chromosome 21q22 is a genetic determinant for the predisposition to rheumatoid arthritis in Japanese population. Human Molecular Genetics. 2011;20(13):2680–5.

10. Hu HJ, Jin EH, Yim SH, et al. Common variants at the promoter region of the APOM confer a risk of rheumatoid arthritis. Experimental & molecular medicine. 2011;43(11):613–21.

11. Negi S, Juyal G, Senapati S, et al. A genome-wide association study reveals ARL15, a novel non-HLA susceptibility gene for rheumatoid arthritis in North Indians. Arthritis and Rheumatism. 2013;65(12):3026–35.

12. Orozco G, Viatte S, Bowes J, et al. Novel rheumatoid arthritis susceptibility locus at 22q12 identified in an extended UK genome-wide association study. Arthritis and Rheumatology. 2014;66(1):24–30.

13. Okada Y, Wu D, Trynka G, et al. Genetics of rheumatoid arthritis contributes to biology and drug discovery. Nature. 2014;506(7488):376–81.

14. Jiang L, Yin J, Ye L, et al. Novel risk loci for rheumatoid arthritis in han chinese and congruence with risk variants in europeans. Arthritis and Rheumatology. 2014;66(5):1121–32.

15. Bossini-Castillo L, de Kovel C, Kallberg H, et al. A genome-wide association study of rheumatoid arthritis without antibodies against citrullinated peptides. Annals of the Rheumatic Diseases. 2015;74(3):e15–e15.

16. Saxena R, Plenge RM, Bjonnes AC, et al. A Multinational Arab Genome-Wide Association Study Identifies New Genetic Associations for Rheumatoid Arthritis. Arthritis & Rheumatology. 2017;69(5):976–85.

17. Wei WH, Viatte S, Merriman TR, et al. Genotypic variability based association identifies novel non-additive loci DHCR7 and IRF4 in sero-negative rheumatoid arthritis. Sci Rep. 2017 Jul 13;7(1):5261.

18. Saad MN, Mabrouk MS, Eldeib AM, et al. Studying the effects of haplotype partitioning methods on the RA-associated genomic results from the North American Rheumatoid Arthritis Consortium (NARAC) dataset. J Adv Res. 2019 Jan 18;18:113-126.

19. Laufer VA, Tiwari HK, Reynolds RJ, et al. Genetic influences on susceptibility to rheumatoid arthritis in African-Americans. Hum Mol Genet. 2019 Mar 1;28(5):858-874.
